# Deamidated Human Triosephosphate Isomerase is a Promising Druggable Target

**DOI:** 10.3390/biom10071050

**Published:** 2020-07-15

**Authors:** Sergio Enríquez-Flores, Luis Antonio Flores-López, Itzhel García-Torres, Ignacio de la Mora-de la Mora, Nallely Cabrera, Pedro Gutiérrez-Castrellón, Yoalli Martínez-Pérez, Gabriel López-Velázquez

**Affiliations:** 1 Grupo de Investigación en Biomoléculas y Salud Infantil, Laboratorio de EIMyT, Instituto Nacional de Pediatría, Secretaría de Salud, Mexico City 04530, Mexico; luisbiolexp@gmail.com (L.A.F.-L.); garcia.itzhel@gmail.com (I.G.-T.); ignaciodelamora@yahoo.com.mx (I.d.l.M.-d.l.M.); 2 CONACYT-Instituto Nacional de Pediatría, Secretaría de Salud, Mexico City 04530, Mexico; 3 Departamento de Bioquímica y Biología Estructural, Instituto de Fisiología Celular, Universidad Nacional Autónoma de México, Mexico City 04510, Mexico; ncabrera@ifc.unam.mx; 4 Hospital General Dr. Manuel Gea González, Mexico City 14080, Mexico; inpcochrane@gmail.com; 5 Unidad de Investigación en Medicina Experimental, Facultad de Medicina, Universidad Nacional Autónoma de México, Mexico City 04510, Mexico; yoalli89@gmail.com

**Keywords:** protein structure, triosephosphate isomerase, Glycolysis, AGEs, SARS-CoV-2, omeprazole, docking

## Abstract

Therapeutic strategies for the treatment of any severe disease are based on the discovery and validation of druggable targets. The human genome encodes only 600–1500 targets for small-molecule drugs, but posttranslational modifications lead to a considerably larger druggable proteome. The spontaneous conversion of asparagine (Asn) residues to aspartic acid or isoaspartic acid is a frequent modification in proteins as part of the process called deamidation. Triosephosphate isomerase (TIM) is a glycolytic enzyme whose deamidation has been thoroughly studied, but the prospects of exploiting this phenomenon for drug design remain poorly understood. The purpose of this study is to demonstrate the properties of deamidated human TIM (HsTIM) as a selective molecular target. Using in silico prediction, in vitro analyses, and a bacterial model lacking the *tim* gene, this study analyzed the structural and functional differences between deamidated and nondeamidated HsTIM, which account for the efficacy of this protein as a druggable target. The highly increased permeability and loss of noncovalent interactions of deamidated TIM were found to play a central role in the process of selective enzyme inactivation and methylglyoxal production. This study elucidates the properties of deamidated HsTIM regarding its selective inhibition by thiol-reactive drugs and how these drugs can contribute to the development of cell-specific therapeutic strategies for a variety of diseases, such as COVID-19 and cancer.

## 1. Introduction

Deamidation of asparagine (Asn) residues is a commonly occurring posttranslational modification in proteins. Deamidation causes de novo negative charges into the protein structure by changing Asn to aspartic acid (Asp) or isoaspartic acid (isoAsp) in a nonenzymatic reaction. In addition, in mammals, such reactions can be directed by N-terminal asparagine amidohydrolase 1 (NTAN1) [1]. This modification is believed to be a major pathway to protein turnover but may also induce structural changes that can lead to new functions [2]. Additionally, deamidation has been associated with aging and Alzheimer disease [3,4,5], among other cellular alterations.

Deamidation at internal Asn residues in proteins occurs near neutral pH through an intramolecular arrangement of two steps. First, the carbonyl carbon of the Asn side chain is attacked by the backbone amide nitrogen atom of the first amino acid residue adjacent to the C-terminal end of Asn, releasing an amide group (deamidation) and forming succinimide. In the second step of the reaction, hydrolysis of succinimide yields Asp or isoAsp at an Asp:isoAsp ratio of 1: 3 [6]. Nonetheless, in some proteins, this ratio can change, resulting in a higher yield of Asp [7].

Deamidation has been thoroughly studied in human proteins such as Bcl-xL [8], the eye lens protein crystallin [9], and the glycolytic enzyme triosephosphate isomerase (TIM) [10]. Information about the TIM deamidation process has accumulated over more than 30 years [11]. Based on what was known about the process, it was demonstrated that TIM deamidation is triggered by two Asn residues at positions 16 and 72 and that the presence of a glycine (Gly) residue next to the C-terminal end of these residues notably increases the deamidation rate [3]. In addition, it is known that continuous catalytic cycles promote such reactions [11], as it has been demonstrated in cellular systems with high levels of glycolytic activity [12]. It is not necessary that both Asn residues in the human TIM (HsTIM) undergo deamidation to show the functional and structural effects of a completely deamidated TIM; instead, deamidation of N16 is sufficient to provoke this [10]. Although several studies have named this phenomenon the terminal marking of TIM for degradation, it has not yet been demonstrated that the deamidated HsTIM has to be degraded in the cell.

The physiologically relevant function described for TIM is the interconversion of glyceraldehyde-3-phosphate (GAP) and dihydroxyacetone phosphate (DHAP) as a central step in the glycolytic pathway, which in turn produces NADH and ATP in cells. DHAP provides a substrate for glycolysis to generate energy, but its accumulation must be avoided because DHAP can be harmful for the cell when it is degraded to methylglyoxal, a highly toxic metabolite [13].

On the other hand, due to the importance of this enzyme to maintain the energy balance in the cell through the glycolytic pathway, TIM has been used as a target for drug design in parasites. On this line, TIMs from parasites such as *Giardia lamblia*, *Trypanosoma cruzi* and *T. brucei*, among others, have been successfully inhibited with cysteine-reactive compounds in a species-specific manner without affecting their enzyme homology in humans [14]. Due to (a) the strong structural destabilization caused by deamidation in HsTIM, which makes it markedly different from its nondeamidated counterpart and (b) the reported increase in deamidated HsTIM in some cellular systems [15,16], we propose that deamidated HsTIM can be a selective molecular target for drug design based on similar inhibition mechanisms as those demonstrated for parasitic TIMs.

Here, we used the recombinant N16D HsTIM mutant to mimic the naturally deamidated TIM and to demonstrate its features as a selective molecular target. In silico analyses showed significant structural changes, such as interface instability and more access to the protein’s inner portion, that are caused by deamidation at position 16. Such structural changes also increased the binding affinity for thiol-reactive compounds and may be responsible for the in vitro inhibition that we demonstrated. Targetable characteristics of the deamidated HsTIM were also demonstrated in situ using *E. coli* Δ*tim* complemented with either wild-type (WT) or N16D HsTIM.

Finally, we propose two main factors participating in the potential druggability of the deamidated HsTIM. First, the accelerated deamidation by increasing glycolytic cycles, and second, the capacity of the deamidated enzyme to propitiate cellular overproduction of methylglyoxal (MGO). Therefore, we should endeavor to search those cells where deamidated HsTIM is accumulating, to study them in light of our proposal. In this regard, some works have recently studied the relationship between glycolysis and SARS-CoV-2 replication, showing that infected monocytes transit to aerobic glycolysis, which facilitates viral replication and the production of soluble mediators that may contribute to lung damage [17]. These monocytes show enhanced glycolysis; then, they might be increasing levels of deamidated HsTIM. Therefore, boosting the production of MGO into these cells by targeting deamidated HsTIM should deserve further studies as a potential therapy for COVID-19, even more considering that glycolysis sustains the SARS-CoV-2 replication and that its proteome is highly labile to MGO.

## 2. Materials and Methods

### 2.1. Reagents and Materials

Luria-Bertani (LB) medium and isopropyl-β-D-thiogalactopyranoside (IPTG) were purchased from VWR Life Science Products (Avantor, Radnor, PA, USA). Glycerol-3-phosphate dehydrogenase (α-GPDH) and reduced nicotinamide adenine dinucleotide (NADH) were purchased from Roche (Penzberg, Upper Bavaria, Germany). Immobilized Metal Affinity Chromatography (IMAC) resin was purchased from Bio-Rad (Hercules, California, USA). Sephadex G-25 Fine Resin was purchased from Amersham Biosciences (Amersham, UK). Amicon Ultra 10 and 30 kDa filters were purchased from Merck-Millipore Corporation (Billerica, Massachusetts, USA). The other reagents that will be mentioned were acquired from Sigma-Aldrich (St. Louis, MO, USA).

### 2.2. In Silico Analysis of the WT and N16D HsTIM Crystallographic Structures

Here, we used the numbering of amino acid residues according to the translated product of the human TIM cDNA (GenBank Accession Number: M10036.1). The WT and N16D HsTIM crystallographic structures that were deposited in the Protein Data Bank (PDB) were subjected to in silico analysis as follows. The atomic coordinates of WT and N16D HsTIM (PDB IDs: 2JK2 and 4UNK, respectively) were submitted to the PDBsum server (PDBsum-EMBL-EBI) to analyze the protein–protein contacts (interface) and tunnel formation (MOLEonline 2.0). For docking studies, Achilles Blind Docking Server (https://bio-hpc.ucam.edu/achilles/) was used, and the structures of WT and N16D HsTIM were analyzed with the sulfhydryl reagent DTNB [5,5′-dithiobis-(2-nitrobenzoic acid)]. All structures were energy minimized with Chimera software [18], and with the resulting new coordinates, docking calculations were carried out with the mentioned server. The docking of DTNB to HsTIM targets was performed without a description of the location of the binding site, and subsequently, only the ligands bound to the interface of the proteins were selected. Finally, the electrostatic potential of the HsTIM structures was determined with the PBEQ Solver server (http://www.charmm-gui.org/?doc=input/pbeqsolver&step=0) [19]; this server calculates the electrostatic potential surface of proteins by solving the Poisson–Boltzmann equation. The crystallographic structures were submitted, and the default values were selected (1.0 for the protein interior constant, 80.0 for the solvent dielectric constant, and 0.15 moles/liter for the salt concentration). The results were loaded and visualized with the Chimera program using a color spectrum ranging from red (−5.0) to blue (+5.0) as the lowest and highest electrostatic potential energy values.

### 2.3. Expression and Purification of Recombinant Enzymes

The WT and deamidated mutant (N16D) genes from HsTIM were cloned into the pET3a-HisTEV vector, as previously reported [10]. The vector provides six histidine (His6) residues and a Tobacco Etch Virus protease (TEVp) recognition sequence at the N-terminus of proteins, which facilitate protein purification. The plasmid plus inserts (pET3a-HisTEV-wt and pET3a-HisTEV-n16d) were transformed into the *Escherichia coli* BL21-CodonPlus (DE3)-RIL strain. Overexpression and purification of recombinant enzymes was performed as previously described [10]. Once purified, they were concentrated with centricons (cutoff of 30 and 10 kDa for WT and N16D HsTIM, respectively) until reaching 0.5 mL, and this process was repeated 3 times by adding 5 mL of 100 mM triethanolamine (pH 7.4) and 10 mM EDTA (TE buffer). Next, the proteins were precipitated with 70% ammonium sulfate and maintained at 4 °C. To remove the His6-TEV tag, the protein suspension was centrifuged at 12,000 rpm for 20 min at 4 °C, and the resulting pellet was resuspended in 50 mM Tris (pH 8.0) and 0.5 mM EDTA and incubated at room temperature for 16 h in the presence of freshly prepared TEVp at 1: 50 (*w*/*w*) (TEVp/HsTIM) with 1 mM dithiothreitol (DTT). At the end of the incubation period, the protein was loaded onto a column with IMAC resin equilibrated with 100 mM triethanolamine (pH 7.4). The enzymes without the His6-TEV tag were concentrated, precipitated with ammonium sulfate and stored at 4 °C until usage. For the assays, the precipitated protein was centrifuged as mentioned, and the pellet was suspended in TE buffer. The protein concentration was calculated spectrophotometrically at 280 nm taking into account an extinction coefficient of the protein (ε = 33,460 M^−1^ cm^−1^) [20]. The purity of the proteins was checked with 16% sodium dodecyl sulfate polyacrylamide gel electrophoresis (SDS-PAGE) and stained with colloidal Coomassie Brilliant Blue. Prior to the assays, the recombinant enzymes were equilibrated in TE buffer and incubated in the presence of 5 mM DTT for 30 min at 4 °C. To remove the reducing agent, the protein was spin filtered in a 1 mL column loaded with Sephadex G-25 Fine Resin equilibrated with TE buffer, after which the protein concentration was spectrophotometrically estimated at 280 nm.

### 2.4. Inactivation Assays of WT and N16D HsTIM with MMTS, MTSES, and DTNB

Assays were performed to explore the inactivation of the enzymes with the sulfhydryl reagents methyl-methanethiosulfonate (MMTS), sodium 2-[(methylsulfonyl)sulfanyl] ethanesulfonate (MTSES) and 5,5′-dithiobis-(2-nitrobenzoic acid) (DTNB). A 1 mM stock of each compound was prepared by dissolving the compound with TE buffer. The recombinant WT and N16D HsTIM enzymes were prepared and incubated at 0.5 mg/mL in the presence of 0, 2.5, 5, 10 and 25 µM of each compound for 2 h at 37 °C. After incubation time, the enzyme activity was measured for each sample diluting to 5 and 50 ng/mL for the WT and N16D HsTIM, respectively. Enzyme activity was spectrophotometrically measured with a Cary 50 Spectrophotometer (Agilent Technologies, Santa Clara, CA, USA) to determine the direction of DHAP synthesis, by employing a coupled system and following the oxidation of NADH at 340 nm [21]. The results were expressed as percent of enzymatic activity versus sulfhydryl reagent concentration, considering the enzymatic activity as 100% of the activity without the compound.

### 2.5. Quantification of Derivatized Cys in WT and N16D HsTIM Treated with Sulfhydryl Reagents

Because the used reagents specifically derivatize Cys residues, the number of derivatized Cys residues was determined in the recombinant enzymes. To achieve this, 0.5 mg/mL of the proteins were incubated separately without or with 250 µM MMTS, MTSES or DTNB for 2 h at 37 °C. After incubation, the samples were extensively washed with centricons to eliminate the excess sulfhydryl, and the enzyme concentration was estimated at 280 nm. Subsequently, the samples were withdrawn and aliquoted to measure the activity of the enzymes. The free thiol content (free Cys) was calculated according to Ellman’s method [22] with modifications. The samples were spectrophotometrically measured considering the basal absorbance at 412 nm of 1 mM DTNB with 5% SDS dissolved in TE buffer. Then, 250 µg of protein was added to the cuvette, and the initial and final absorbance was monitored. The content of Cys was calculated by taking into account the extinction coefficient of DTNB (ε = 14.1 mM^−1^ cm^−1^). Finally, the number of derivatized Cys was obtained by subtracting the free Cys of the derivatized enzymes (exposed to sulfhydryl compounds) from the free Cys of the enzymes in the absence of the compounds (control).

### 2.6. Growth and Inhibition Curves of E. coli Δtim-BL21-Gold(DE3) Cells Complemented with WT and N16D Hstim Genes

To determine the biological significance of N16D HsTIM (deamidated enzyme) and demonstrate that it is possible to selectively direct thiol reagent drugs to this protein under the cellular environment, the *E. coli* BL21-Gold(DE3) strain, which does not contain the tim gene (*E. coli* Δ*tim*), was employed [23]. The cells were grown in M9 minimal medium plus 100 μg/mL ampicillin. For cell growth, 1.5 mL flat-bottom plates (CLS3526 24-well plates, Corning Costar) were used. All assays were performed in triplicate. In a total volume of 1.5 mL, a single colony of complemented bacterial cells was inoculated with the plasmid (pET-3a-His-TEVp) plus the insert of either WT or N16D HsTIM. Growth was followed for 22 h at 37 °C under gentle shaking, and the absorbance was monitored at 600 nm with Synergy MX equipment (BioTek, Winooski, VT, USA). For the inhibition growth assays, omeprazole was used as a thiol-reactive drug, and an aliquot of a stock solution of 400 mM omeprazole dissolved in dimethyl sulfoxide (DMSO) was added to the cultures at time zero. The final concentrations of omeprazole and DMSO were 0.75 mM and 0.18%, respectively. The cultures were incubated under agitation for 22 h, and the absorbance of bacterial growth was measured spectrophotometrically at 600 nm. The results were monitored with Gen 1.11 software. Next, at the end of the experiment, the cells were centrifuged, and the pellet was resuspended in 50 mM Tris (pH 8.0) and 100 mM NaCl. Then, the bacteria were lysed by ten freeze/thawing cycles and centrifuged at 9000 rpm for 20 min at 4 °C; to determine HsTIM activity, as previously mentioned, the results of cellular growth were expressed in terms of absorbance versus time (h), and the HsTIM activity was expressed as the percent of enzymatic activity (WT or N16D HsTIM) in the absence or presence of omeprazole.

### 2.7. Methylglyoxal and AGE Quantification in E. coli Δtim-BL21-Gold(DE3) Cells Complemented with WT and N16D Hstim Genes

It has been widely described that DHAP (one of the TIM substrates) accumulates when the glycolytic enzyme is functionally inhibited [24]; in turn, this substrate degrades to a highly toxic metabolite known as methylglyoxal (MGO). Therefore, the MGO was quantified as follows. *E. coli* Δtim-BL21-Gold(DE3) cells complemented with either WT or N16D HsTIM were grown at 37 °C under agitation for 22 h, with or without 0.75 mM omeprazole. At the end of the incubation time, the cells were centrifuged at 2500 rpm for 20 min at 4 °C, and the pellet was resuspended in 50 mM Tris (pH 8.0) and 100 mM NaCl. The bacteria were lysed by ten freeze/thaw cycles and centrifuged at 9000 rpm for 20 min at 4 °C, and then 0.45 M perchloric acid was added to the supernatant of each sample, which was incubated on ice for 10 min and centrifuged at 12,000 rpm at 4 °C for 10 min. Next, the supernatant was collected and stored at −70 °C for further measurement. To quantify the free MGO from bacterial cells, standard values of MGO were determined according to the method described by Gilbert and Brandt [25] with modifications [26]. Stock solutions of 20 mM 2,4-dinitrophenylhydrazine (DNPH) in HCl-ethanol (12:88) and 1 mM MGO in distilled water were prepared. For the assays, increasing concentrations of MGO (0 to 10 μM) were incubated with 0.2 mM DNPH at 42 °C for 45 min, after which the absorbance of methylglyoxal-bis-2,4-dinitrophenylhydrazone was spectrophotometrically measured at 432 nm. In parallel, the stored supernatants of the *E. coli* Δtim cells were used to quantify intracellular MGO. Finally, MGO concentrations from the standard curve and cells were estimated, taking into account the extinction coefficient of methylglyoxal-bis-2,4-dinitrophenyl-hydrazone (ε = 33,600 M^−1^ cm^−1^). The results were expressed as nmol of MGO/mL.

Because MGO is irreversibly bound to DNA and proteins (mostly to proteins) [27], advanced glycation end products (AGEs) were measured with the AGE ELISA Kit (MBS267540, MyBioSource, San Diego, CA, USA), according to the manufacturer’s instructions with modifications [26]. A precoated antibody was used as the AGE monoclonal antibody, and the detection antibody was a biotin-labeled polyclonal antibody.

From the experimental assays mentioned above to determine MGO, aliquots of supernatants from lysed and centrifuged cells (*E. coli* Δ*tim*-BL21-Gold(DE3) complemented with WT or N16D HsTIM, treated without and with omeprazole) were collected, and the protein concentration was determined with a bicinchoninic acid assay. Standard values were determined with standard AGE samples at the following concentrations: 200, 100, 50, 25, 12.5, 6.25, and 3.12 ng/mL. In parallel, samples of *E. coli* lysates were diluted to a concentration of 1 μg/μL, subsequently diluted at a ratio of 1:100 and loaded into the ELISA plate for the determination of the AGE concentration, following the manufacturer’s instructions. The optical density (OD) at 450 nm was measured on a microplate spectrophotometer (EPOCH, BioTek, Winooski, VT, USA).

### 2.8. Cellular Assays with E. coli BL21-CodonPlus (DE3)-RIL in the Presence of Omeprazole

To identify the interaction of the thiol drug with the target protein, assays were conducted with the *E. coli* BL21-CodonPlus (DE3)-RIL strain (Agilent Technologies, Santa Clara, CA, USA) that contained either the pET3a-HisTEV plasmid with the WT or N16D HsTIM insert or the plasmid without an insert. Cells were grown in LB medium supplemented with ampicillin (100 µg/mL) and chloramphenicol (50 µg/mL), as mentioned above, until reaching an OD of 1, which was spectrophotometrically measured at 600 nm. Next, DMSO (control) or 0.75 mM omeprazole was added to the cultures, which were incubated at 30 °C for 12 h. The following day, the cells were centrifuged, and the pellets were resuspended in 50 mM Tris (pH 8.0) and 100 mM NaCl and lysed by sonication. Cell lysates were centrifuged at 9000 rpm for 30 min at 4 °C, and the supernatant was used to determine the protein concentration with the bicinchoninic acid assay. Finally, the samples were loaded onto an SDS-PAGE gel; the gel was visualized using the ChemiDoc XRS+ System (Bio-Rad) (to detect the omeprazole signal) and thereafter stained with Coomassie Brilliant Blue.

### 2.9. Statistical Analysis

All results are expressed as the means ± standard deviations (SD). All data were analyzed using the GraphPad Prism statistical software package program (Ver. 8.4.2). Statistical comparisons were performed using one-way analysis of variance (ANOVA) followed by Dunnett’s test. *p* values < 0.05 were considered statistically significant.

## 3. Results

### 3.1. Deamidation Alters the Interatomic Interacting Network in HsTIM

**Figure 1:**
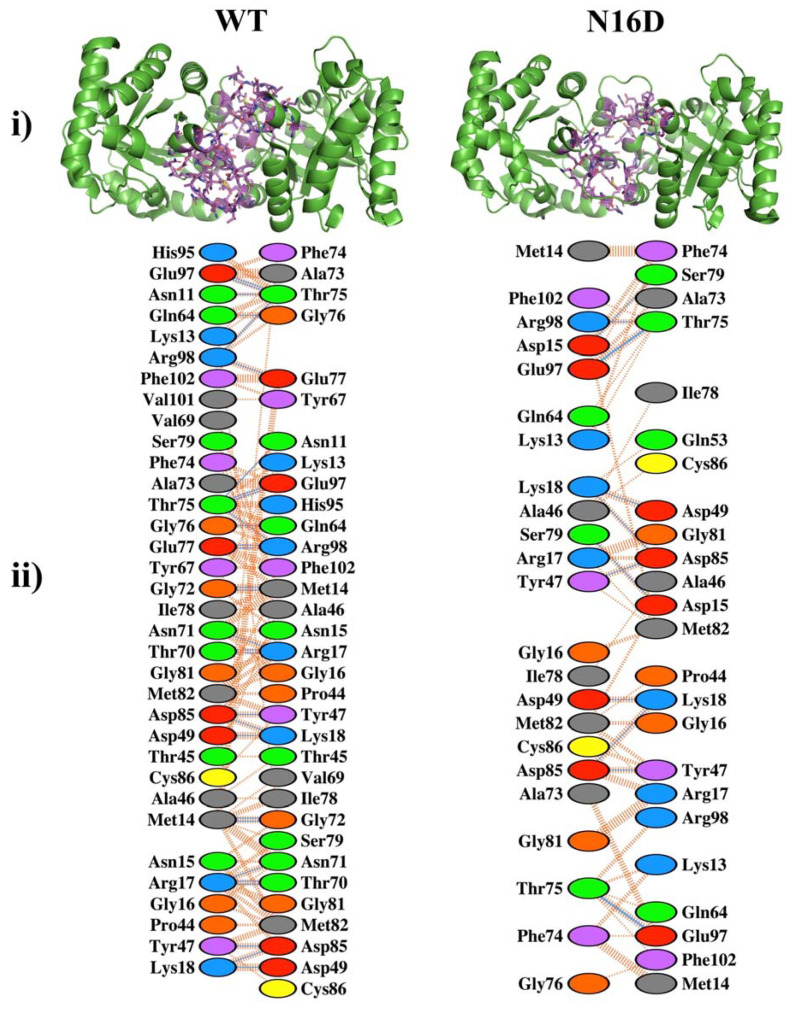
Noncovalent interactions in the interfaces of the nondeamidated (WT) and deamidated (N16D) human triosephosphate isomerase (HsTIM) based on their crystallographic structures. (**i**) The green cartoon is both subunits (dimer) of the WT and N16D HsTIM. The amino acids involved in the noncovalent interactions at the interfaces are shown in purple sticks. (**ii**) The amino acids and their interatomic contacts are depicted at the interfaces of both subunits. Figures were modeled with (**i**) PyMOL [28] and (**ii**) PDBsum-EMBL-EBI [29]. In (**ii**), the color code of ovals represents the properties of the side chain of the amino acids: positive (blue); negative (red); neutral (green); aliphatic (gray); aromatic (violet); proline (brown) and cysteine (yellow).

Since deamidation occurs close to the contact site of the two adjacent subunits in HsTIM (interface), we initially compared the interactions established between the amino acid residues that conform to this region in nondeamidated (WT) and deamidated (N16D) HsTIM. The number of total amino acid contacts were 319 and 164 for WT and N16D HsTIM, respectively (Figure 1 and Supplementary Table S1). The interface of N16D HsTIM decreased its interatomic contacts by 48.5% and, consequently, lost 27.48% of the contact area in both subunits with respect to that of the WT HsTIM (1710 and 1240 A^2^ for WT and N16D HsTIM, respectively). These results indicate that the incorporation of de novo negative charges in N16D HsTIM leads to important structural alterations in the interatomic network of contacts at the interface of the enzyme.

**Figure 2:**
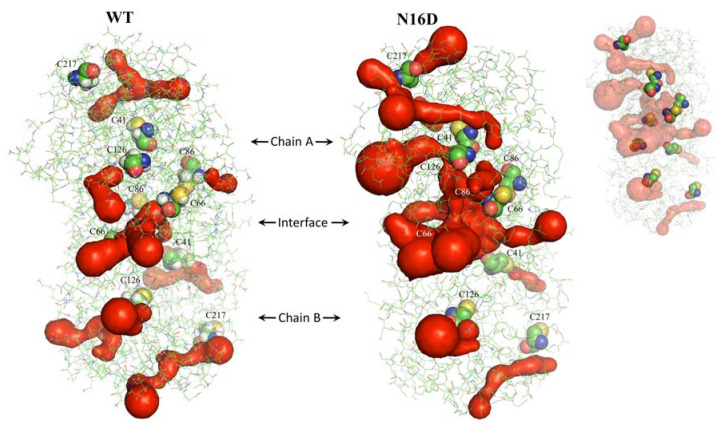
Identification of tunnels in WT and N16D HsTIM. Dimers of both crystallographic structures are shown as green lines, and the surface of tunnels is shown in red. Cys residues are represented with spheres, and the inset shows those hidden in the tunnels. These structures were analyzed with MOLEonline [30] and prepared with PyMOL [28].

Deamidation of HsTIM also elicited effects by perturbing noncovalent interactions between its constituent amino acids inside the protein. Consequently, the common 14 tunnels or “galleries” found in WT HsTIM increased to 26 in N16D HsTIM (calculated with the MOLEonline web interface). Additionally, the tunnels of N16D HsTIM are longer than those in WT HsTIM and are closer to the Cys residues (Figure 2, Supplementary Table S2). These results show high permeability in the structure of N16D HsTIM and strongly suggest greater accessibility of the solvent and small molecules to the core of the protein; thus, previously buried amino acid residues (i.e., Cys) in WT HsTIM may be targetable.

### 3.2. Binding Sites for Thiol-Reactive Compounds Are Increased into the Interface of N16D HsTIM

**Figure 3:**
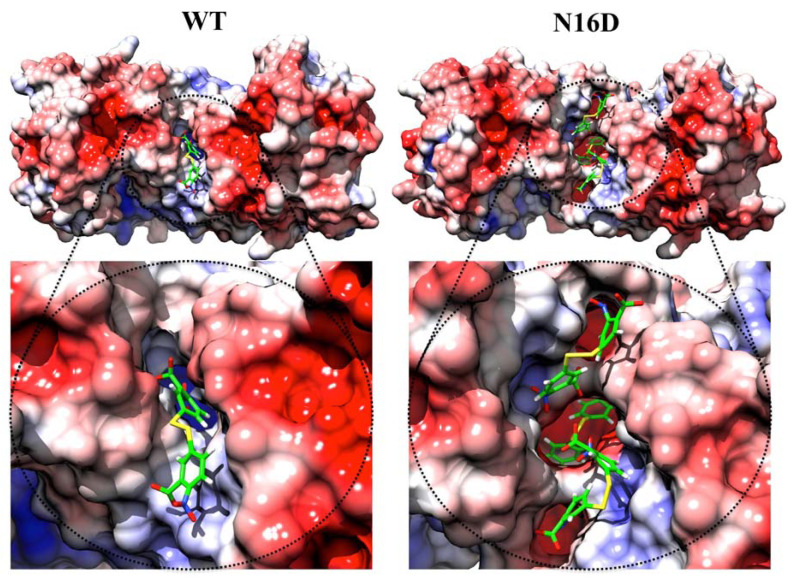
Docking of 5,5′-dithiobis-(2-nitrobenzoic acid) (DTNB) and electrostatic potential surface of the WT and N16D HsTIM structures. The figures are an ensemble of docking and the electrostatic potential surface results. As seen in WT HsTIM, DTNB was incorporated superficially in the interface of this structure, while in N16D HsTIM, three DTNB molecules were docked in the same region. Moreover, one DTNB was successfully docked in the innermost zone of the interface in N16D HsTIM. As seen in the electrostatic potential near the DTNB in WT or N16D HsTIM, positive charges are confirmed. However, in the innermost zone of the interface in N16D HsTIM, the negative charges conform. Color codes represent the electrostatic potential surface energy values of −5.0 (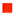) and +5.0 (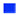). Figures were modeled with the molecular graphics images produced with the UCSF Chimera package [18].

In silico analysis predicted an increase in the binding sites for DTNB (a thiol-reactive compound) into the interface of N16D HsTIM with respect to the interface in WT HsTIM (Figure 3). Additionally, a variety of conformers of this molecule were suitable to bind the interface (Supplementary Figures S1 and S2). It is important to note that the interface of N16D HsTIM shows more binding sites than that in WT HsTIM, and some of these binding sites were the deepest found in both structures (Supplementary Figures S1 vs. S2). These results strongly suggest that small molecules could have reached the inner portion of N16D HsTIM and selectively targeted some amino acids in the enzyme that are hidden in the case of the WT HsTIM protein.

Additionally, the electrostatic potential surface calculated for both structures shows a slight positive electrostatic potential for the interface of both structures (Figure 3). Such electrostatic features could facilitate the attraction of molecules with opposite charges (i.e., negatively charged as DTNB). However, the core of the interfacial region in N16D HsTIM shows a prevalence of negative charges (Figure 3, right), which could represent a factor that contributes to the destabilization of the enzyme through this region. Therefore, we performed in vitro assays to direct thiol-reactive molecules such as DTNB against HsTIM and to demonstrate that the Cys residues in N16D HsTIM are selectively targeted in comparison with those in WT HsTIM.

### 3.3. The N16D HsTIM Enzyme Is Totally and Selectively Inactivated with Thiol-Reactive Compounds

**Figure 4:**
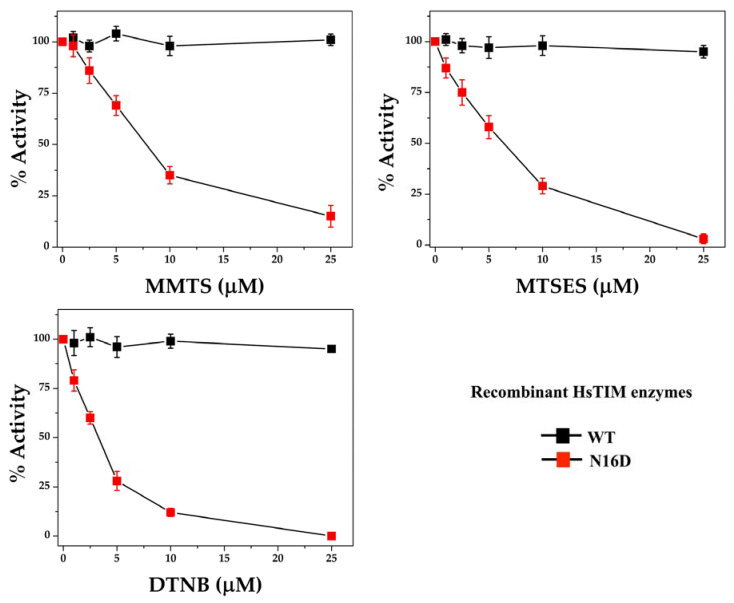
Inactivation assays of recombinant WT and N16D HsTIM enzymes. WT and N16D HsTIM were incubated at 0.5 mg/mL with 0, 1, 2.5, 5, 10 and 25 µM methyl-methanethiosulfonate (MMTS), sodium 2-[(methylsulfonyl)sulfanyl] ethanesulfonate (MTSES) or DTNB. After the incubation time, aliquots were withdrawn at each experimental condition and assayed for their enzymatic activity. Enzyme activity was assayed using 5 and 50 ng mL of WT and N16D HsTIM, respectively.

Increasing concentrations of three different thiol-reactive compounds, MMTS, MTSES, and DTNB, were assayed in vitro against the WT and N16D HsTIM enzymes. All of these thiol-reactive compounds lead to the total inactivation of N16D HsTIM, whereas under the same conditions, the WT HsTIM retains its original enzyme activity (Figure 4).

The enzyme activity of N16D HsTIM dropped to 50% with 7.5, 6, and 3 µM MMTS, MTSES, and DTNB, respectively. Although the differences in the inactivation between these compounds were marginal, DTNB was the most efficient compound to inhibit N16D HsTIM. Therefore, these results support our in silico analyses and experimentally demonstrate that the N16D HsTIM enzyme might be selectively druggable by targeting its Cys residues.

The role that the chemical modification of Cys residues (derivatization) plays in the inactivation of N16D HsTIM was tested by quantifying the number of derivatized Cys with each of the thiol-reactive compounds. While the WT HsTIM showed only 1 derivatized Cys/subunit (HsTIM contains 5 Cys/subunit) at the highest concentrations of each thiol-reactive compound, the N16D HsTIM reached 4 derivatized Cys/subunit (Table 1).

Therefore, these results demonstrate that the Cys residues of N16D HsTIM are derivatized with thiol-reactive compounds and that this process inactivates N16D HsTIM in a selective and efficient manner.

### 3.4. E. coli Δtim Cells Complemented with the WT and N16D Genes Are a Good Model to Study the Effects of N16D HsTIM at the Cellular Level

**Figure 5:**
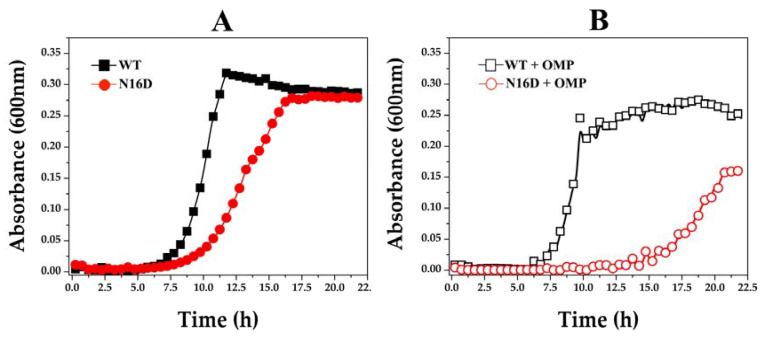
Growth curves of *E. coli* Δ*tim* complemented with WT and N16D Hstim genes. The *E. coli* strain Δ*tim* BL21-Gold(DE3) was transformed with the overexpression plasmid pET-3a HisTEV, encoding either WT or N16D HsTIM. In (**A**), the growth of cells supplemented with WT (filled black squares) and N16D (filled red circles) HsTIM in the absence of omeprazole is shown. In (**B**), the growth of cells supplemented with WT (open black squares) and N16D (open red circles) HsTIM in the presence of 0.75 mM omeprazole is shown. The optical density (OD600) was plotted against the incubation time (h) in M9 minimal medium supplemented at 37 °C. The assays were performed in quadruplicate on a Synergy MX microplate reader on 96-well plates.

A druggable target must be accessible to the putative drug molecule, and the used drug has to be safe. Although the bacterial model is not appropriate when designing the study for drug-target interaction, it is useful to understand the affectation of HsTIM into the cell milieu. Thus, we performed a series of assays using *E. coli* Δ*tim* BL21-Gold(DE3), a genetically manipulated *E. coli* strain without the *tim* gene [23], complemented with either WT or N16D HsTIM. Additionally, based on the inhibition mechanisms described above and following the principles of drug discovery, we used omeprazole, a safe, thiol-reactive drug. Figure 5 shows the growth curves of *E. coli* Δ*tim* complemented with the assayed *Hstim* genes. Cells transfected with the plasmids overexpressing WT HsTIM reached their highest growth rate after incubation for 11 h, whereas those with N16D HsTIM delayed the growth of the Δ*tim* bacterial strain. Nonetheless, *E. coli* Δ*tim* complemented with N16D HsTIM also reached the maximal growth rate reached by WT HsTIM, but 5 h later (Figure 5A). After confirming that the growth of *E. coli* Δ*tim* is successful when they are complemented with both genes, we performed assays culturing these complemented cells in the presence of omeprazole and followed their growth (Figure 5B).

Under the described conditions, the growth curves of *E. coli* Δ*tim* cells complemented with WT HsTIM were almost the same, both in the absence and presence of omeprazole (Figure 5A,B, respectively). Conversely, the cells complemented with N16D HsTIM notably had a delayed and impaired growth when exposed to omeprazole (Figure 5B). In addition, these cells never reached the maximum growth rate (Figure 5B) that was observed in the absence of omeprazole (Figure 5A).

These results strongly suggest that the inactivation exerted by omeprazole on N16D HsTIM is responsible for the observed growth impairment. Consequently, we assayed the enzyme activity of HsTIM on the complemented bacterial cells. The results demonstrated that the TIM activity in cells complemented with WT HsTIM was almost the same both in the absence and presence of omeprazole, whereas the TIM activity in the cells complemented with N16D HsTIM was totally abolished in the presence of omeprazole (Table 2).

### 3.5. Omeprazole Induces Increasing Levels of MGO and AGEs in E. coli Δtim Cells Complemented with N16D HsTIM

The in situ inactivation of N16D HsTIM with a thiol-reactive drug such as omeprazole might be linked to the accumulation of TIM metabolites, as occurred in other cell models [26]. This is the case for DHAP, one of the substrates of TIM, which in turn can be spontaneously degraded to the toxic metabolite MGO. Therefore, we quantified the MGO concentration in *E*. *coli* Δ*tim* cells complemented with *Hstim* genes. In this experiment, the concentration of MGO in cells complemented with WT HsTIM was 580 nmol/mL (which was set as 100%), whereas the presence of omeprazole increased this value by only 19% (Table 3). On the other hand, the cells complemented with N16D HsTIM showed 1,506 nmol/mL MGO in the absence of omeprazole (2.6-fold) and reached 2,617 nmol/mL MGO when this drug was present, which corresponded to a 4.5-fold increase in MGO in N16 HsTIM compared to the WT HsTIM control (Table 3).

**Figure 6:**
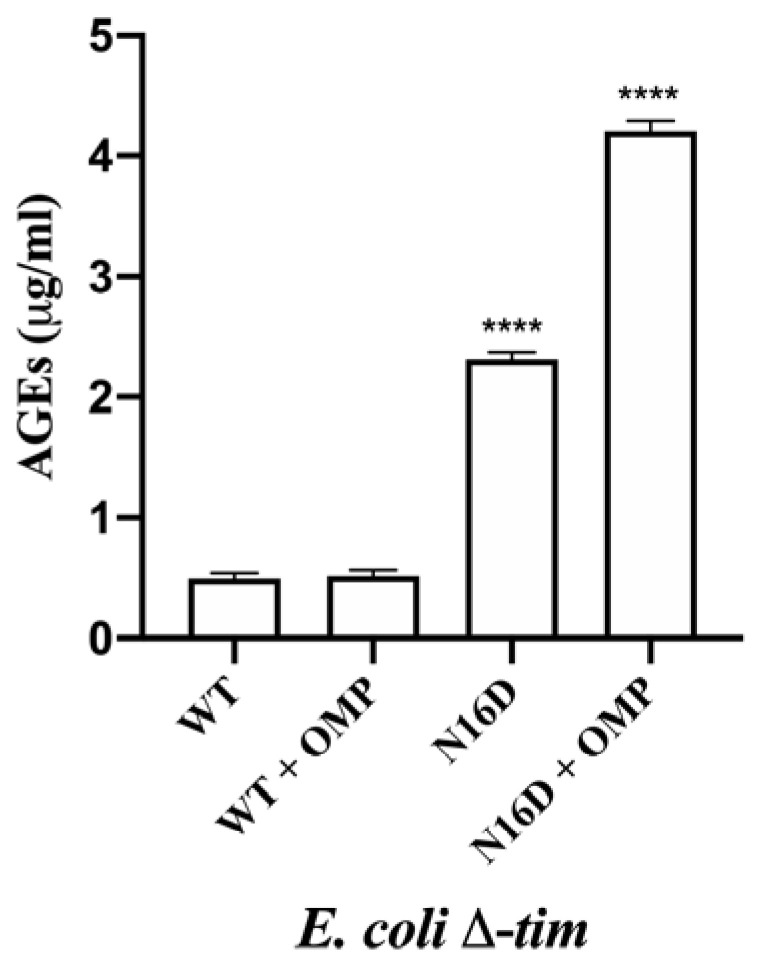
Quantification of AGEs in *E. coli* Δ*tim* cells complemented with WT and N16D HsTIM in the absence or presence of 0.7 mM omeprazole (+ OMP). After incubating the cells in the absence or in the presence of OMP, cells were subjected to the determination of advanced glycation end products (AGEs) with anti-AGE antibodies, as described in the Materials and Methods. The results are presented as the mean ± standard error of three independent experiments. Ordinary one-way analysis of variance (ANOVA) followed by Dunnett’s test showed significant differences between WT (with or without OMP) and N16D HsTIM (both with and without OMP). Additionally, significant differences between N16D HsTIM and N16D HsTIM + OMP were found; **** statistically significant differences between groups *p* < 0.0001.

An important consequence of the accumulation of MGO is that it covalently binds to biopolymers, and the adducts further rearrange into stable modifications known as AGEs, which in turn damage cell lipids and proteins [31,32]. Therefore, we tested *E. coli* Δ*tim* cells complemented with the mentioned genes to corroborate the apparition rates of AGEs. Cells were incubated without or with 0.7 mM omeprazole for 22 h, and the concentrations of AGEs were measured. The cells complemented with WT HsTIM showed similar levels of AGEs at any condition, whereas the cells complemented with N16D HsTIM showed significantly higher levels of AGEs (Figure 6). Moreover, these latter cells markedly showed an increase in AGEs, and this increase was highly significant when omeprazole was added (Figure 6).

These results are concordant with those of the MGO production and might be interpreted as a direct consequence of the inactivation process that N16D HsTIM underwent by using omeprazole. Finally, based on the capacity of omeprazole to emit fluoresce after exposure to UV light [33], we demonstrated that HsTIM is targeted into *E. coli* BL21-CodonPlus-RIL cells. Thus, SDS-PAGE without staining show the fluorescence of HsTIM-omeprazole adducts demonstrating that omeprazole reaches HsTIM in the transformed *E*. *coli* BL21-CodonPlus-RIL cells and that N16D HsTIM is markedly more targeted than its WT HsTIM counterpart (Supplementary Figure S4). Moreover, the activities of HsTIM were similar to those obtained with *E. coli* Δ*tim* cells, and the activity of bacterial TIM was poorly affected (Supplementary Table S3).

## 4. Discussion

### 4.1. The Structural Differences between Deamidated and Nondeamidated HsTIM Are the Keystone to Being a Targetable Molecule

Previously, it was demonstrated that N16D HsTIM resembles the native TIM deamidated at position 16, showing structural differences with its nondeamidated counterpart (WT HsTIM) [10]. Herein, we demonstrated the structural changes underwent by deamidation in HsTIM, which drastically perturbs the noncovalent interactions into the interface (Figure 1). The importance of the interfacial interactions in maintaining the association between the two subunits of HsTIM is known to lead to the structural stability needed to support the optimal catalytic function of the enzyme [34,35,36].

Protein deamidation causes changes in the charge and conformation of proteins [3]; in addition to the interfacial alterations described above for N16D HsTIM, we found an extended gallery of tunnels communicating with the solvent within the protein (Figure 2). Both the aperture of the interface and the de novo tunnel formation establish a condition of susceptibility due to the accessibility of amino acid residues that were previously buried and inaccessible. TIM inactivation has further been demonstrated by Cys modification in TIMs with Cys residues accessible to thiol-reactive compounds [37,38,39]. Thus, the interfacial Cys is essential for dimer dissociation by promoting enzymatic inactivation [40]. The latter is the main reason for WT HsTIM resistance to inactivation by the Cys derivatization mechanisms because this enzyme has a methionine (Met) residue instead of Cys in the interfacial region [39]. Our in silico analyses suggest the appearance, in the deamidated enzyme, of open gates toward some buried Cys residues as a consequence of the tunnel formation (Figure 2). Similar to deamidation, the inclusion of de novo negative charges in proteins can direct structural changes, leading to functional implications. For example, the glucose-6-phosphate dehydrogenase (G6PD) Yucatan variant has a negative charge in its structure due to the K429E mutation, which increases the instability by affecting the interactions with the structural NADP^+^ and neighboring amino acids, resulting in one of the most severe clinical phenotypes of this deficiency [41]. Indeed, by performing Fourier-transform ion cyclotron resonance tandem mass spectrometry (FTICR-MS) and computational flexibility analysis, the global structural affectation promoted by deamidation was demonstrated on calmodulin and β-2-microglobulin [42].

### 4.2. Deamidated TIM Is More Permeable to Thiol-Reactive Compounds than Its Nondeamidated Counterpart

It is well known that WT HsTIM is resistant to thiol-reactive compounds due to the poor accessibility to the Cys residues in this enzyme [14,39]. Based on the identification of the striking aperture of the interface and other regions in the N16D HsTIM enzyme, we postulated the possibility of directing thiol-reactive compounds to those regions. The N16D HsTIM incorporated a major number of DTNB molecules, which also docked deeper in the structure than those in the WT HsTIM (Figure 3). This larger permeability to the compounds could be a consequence of the new steric conditions shown by the enzyme in conjunction with its electrostatic features.

Since molecular docking based on X-ray crystallography structures plays an important role in structure-based drug design [43,44], we took these findings as the basis to perform biochemical studies to demonstrate our in silico predictions. Other studies, such as those on viral neuraminidase, have used similar strategies with good results [45,46]. Therefore, as WT HsTIM has already been demonstrated to be unaffected by thiol-reactive compounds [14], it is a good model to contrast with the in silico prediction of the possible effects in N16D HsTIM by such compounds.

### 4.3. Thiol-Reactive Compounds Selectively Affect N16D HsTIM

By using thiol-reactive compounds (Supplementary Table S4), which promote the formation of adducts with different chemical and volume characteristics [14,47], N16D HsTIM was highly sensitive to all of them, whereas WT HsTIM was not. Nonetheless, DTNB (negatively charged and with the largest volume) exerted a slightly stronger effect than the other compounds (Figure 4). Importantly, the differential inactivation of N16D HsTIM is based on a Cys modification, as shown by the quantification, where of the 5 Cys residues per monomer, WT HsTIM shows only 1 modified Cys, whereas N16D HsTIM shows 4 modified Cys residues (Table 1). The well-documented chemoselectivity of the assayed compounds toward Cys to form disulfides [48] supports our claim to propose that the Cys residues of N16D HsTIM are drug design targets. It is important to note that TIM from several parasites is naturally sensitive to such compounds [14,37,38,49,50,51], but HsTIM is strictly affected after being deamidated. Although the in silico analyses did not show an evident exposition of the Cys residues in N16D HsTIM, the in vitro assays were met and far exceeded the computational predictions on the permeability of this enzyme. Functional results previously demonstrated the increased accessibility to Cys that is naturally hidden in WT HsTIM. This is because the computational analyses were performed exclusively from one conformational stage solved from the crystallographic structure; it is that protein dynamics in the solution play an important role in breaking through to the core of the protein.

### 4.4. N16D HsTIM Is the Intracellular Druggable Target

Knockout *E. coli* cells are a validated model for rating the effect of heterologous proteins on cell growth [23,52]; therefore, based on our results, we conclude the following: (1) Cell growth complemented with N16D HsTIM is slower than that complemented with WT HsTIM. (2) Both N16D and WT HsTIM complementation reach the same maximal growth density. (3) The thiol-reactive drug does not affect cell growth in WT HsTIM-complemented cells. (4) The thiol-reactive drug affects cell growth in N16D HsTIM-complemented cells. (5) The thiol-reactive drug reaches the intracellular heterologous HsTIM. (6) The thiol-reactive drug does not affect the endogenous *E. coli* TIM.

The slowed growth observed in N16D HsTIM-complemented *E. coli* Δ*tim* cells should be a consequence of the diminished energetic supply, which is supported by the lower enzyme activity shown by N16D HsTIM than by WT HsTIM [10]. Nonetheless, since these cells reach the maximal cell density observed in those complemented with WT HsTIM, it is obvious that energetic yield is not the factor that controls the cell death process that we observed when the thiol-reactive drug is added.

On this basis, it is known that impairment of TIM activity results in the accumulation of DHAP followed by its chemical degradation into the toxic MGO, leading to the formation of AGEs [10,53]. Therefore, the increased levels of AGEs observed in N16D HsTIM-complemented cells treated with omeprazole (Figure 6) are a consequence of the enzymatic effects caused by the thiol-reactive drug on this protein. Therefore, the increase in AGEs over the threshold levels normally shown by cells complemented with WT or N16D HsTIM causes cell death.

Finally, the overall results are in agreement with those obtained from complemented bacterial cells, demonstrating that deamidated HsTIM (N16D) is a good candidate for drug design in the context of a living cell.

## 5. Conclusions

The results presented herein should be an opportunity to take advantage of designing new strategies against a variety of diseases. Thus, an urgent requirement is to search for new treatments for COVID-19. The SARS-CoV-2 proteome shows a 5-fold enrichment of MGO modification sites compared to the human host, which in turn indicates selective toxicity of MGO to the virus. Very recently, by using antitumor agents, doxorubicin and paclitaxel, Thornalley et al. demonstrated that the effect of these drugs is linked to increased glucose metabolism and related increased formation of MGO. They proposed their findings as evidence of vulnerability of SARS-CoV-2 to inactivation by MGO and as a scientific rationale for repurposing these antitumor agents for treatment of COVID-19 [54].

In light of our findings, HsTIM might play a central role in the proposal of Thornalley’s group. Since doxorubicin and paclitaxel increase glycolytic cycles, a likely explanation of the accumulation of MGO would be linked to the inherent increase in deamidated HsTIM, as we presented here. Accordingly, it seems promising to search for a new treatment against COVID-19 based on the use of either doxorubicin or paclitaxel combined with omeprazole to boost the intracellular production of MGO, thereby inactivating the vulnerable proteins of the virus.

Our work is innovative in the way that the concept of molecular targets is shown and opens new expectations in the field of drug design, facing the challenge of current and future diseases. Therefore, human cells, which accumulate deamidated HsTIM, should be targeted by thiol-reactive drugs, as shown herein. Such conditions could be found in highly proliferating, aging, and highly glycolytic cells. Currently, we are further studying the efficacy of our proposal in tumoral models.

## Figures and Tables

**Table 1:** Cys quantification in the WT and N16D HsTIM enzymes.

Enzyme	Thiol-Reactive Compound	Free Cys/Subunit	Derivatized Cys/Subunit
WT	Control *	5.1 ± 0.2	0
	+ MMTS	3.9 ± 0.3	1
	+ MTSES	4.1 ± 0.2	1
	+ DTNB	3.8 ± 0.3	1
N16D	Control *	4.8 ± 0.4	0
	+ MMTS	0.9 ± 0.3	4
	+ MTSES	1.1 ± 0.4	4
	+ DTNB	0.8 ± 0.3	4

* Enzyme without thiol-reactive compound.

**Table 2:** Triosephosphate isomerase (TIM) activity in *E. coli* Δ*tim* cells complemented with WT and N16D HsTIM.

*E. coli* Δ*tim* Cells Complemented with HsTIM	Condition	Enzyme Activity (%)	Enzyme Activity (µmol/min mg)
WT	Control *	100	165 ± 11
	+ Omeprazole	96 ± 4	158 ± 4
N16D	Control *	100	3.13 ± 0.045
	+ Omeprazole	1.95 ± 0.7	0.061 ± 0.023

* Cells without omeprazole.

**Table 3:** Methylglyoxal (MGO) determination from *E. coli* Δ*tim* cells complemented with WT and N16D HsTIM.

*E. coli* Δ*tim* Cells Complemented with HsTIM	Condition	MGO (nmol/mL)	MGO (%)
WT	Control *	580	100
	+ Omeprazole	694 ± 42	119 ± 6 **
N16D	Control *	1506 ± 76	259 ± 5 **
	+ Omeprazole	2617 ± 183	451 ± 7 **

* Cells without omeprazole; ** Percentages with respect to the WT HsTIM control.

## Supplementary Materials

The following are available online at https://www.mdpi.com/2218-273X/10/7/1050/s1, Figure S1: Docking of DTNB and electrostatic potential surface of HsTIM WT structure, Figure S2: Docking of DTNB and electrostatic potential surface of HsTIM N16D structures, Figure S3: Scavenger effect of β-mercaptoethanol on omeprazole sulfenamide, Figure S4: Fluorescence of HsTIM-omeprazole adducts, Table S1: Non-covalent interactions of the interfaces in the crystallographic WT and N16D HsTIM structures, Table S2: Tunnel parameters of the crystallographic structures of WT and N16D HsTIM, Table S3: TIM activity determination from *E. coli* BL21-CodonPlus-RIL cells with WT and N16D HsTIM or without gene insert, Table S4: Characteristics of the thiol-reactive compounds.
