# Supplementary material for: Deamidated Human Triosephosphate Isomerase is a Promising Druggable Target
Source: Biomolecules. 2020 Jul 15;10(7):1050. doi: 10.3390/biom10071050 (PMC7407242; doi:10.3390/biom10071050)
Supplement: Supplementary file 1 [file biomolecules-10-01050-s001.pdf]

SUPPLEMENTARY MATERIALS.

Supplementary Table S1. Non-covalent interactions of the interfaces in the crystallographic WT and N16D HsTIM structures.

HsTIM	Chain	Interface residues	Interface area (Å^2)	Hydrogen bonds	Salt bridges	Non-bonded contacts
WT	A	34	1710	29	4	286
	B	33	1710			
N16D	A	23	1241	13	2	149
	B	24	1224			

* Data generated in PDBsum. PDBsum EMBL-EBI (Laskowski RA (Jan 2001). "PDBsum: summaries and analyses of PDB structures" Nucleic Acids Research. **29** (1): 221–2)

Supplementary Table S2. Tunnel parameters of the crystallographic structures of WT and N16D HsTIM.

Number of Tunnel WT HsTIM (PDB ID: 2jk2)	Length of the channel (Å)	Radius of channel bottleneck (Å)	Number of Tunnel N16D HsTIM (PDB ID:4unk)	Length of the channel (Å)	Radius of channel bottleneck (Å)
1	17.2	1	1	7.9	1.5
2	18.6	1.4	2	12.3	1
3	21.9	0.9	3	18.7	2
4	22.8	1.3	4	20.1	1.9
5	25.5	1.3	5	20.4	1.5
6	28.8	0.9	6	21.8	0.9
7	33	0.9	7	25.6	1.5
8	9.8	1.4	8	25.9	1.1
9	10.7	1.3	9	27	1.1
10	12.9	1.4	10	28.5	1.4
11	15.8	1.3	11	32.4	1.5
12	27.7	1	12	34.4	1.3
13	12.3	1.2	13	36.8	0.9
14	13.1	0.8	14	38.1	1.1
			15	47	0.9
			16	47.9	1.1
			17	9.5	1.3
			18	13.3	1.4
			19	15.2	1.2
			20	24.5	1.3
			21	18.3	1.2
			22	20.6	1.2
			23	8.9	1.2
			24	9.8	1.4
			25	5.7	1.9
			26	8	1.7

* Data generated with MoleOnline. MOLE*online* (Pravda L, Sehnal D, Toušek D, Navrátilová V, Bazgier V, Berka K, Svobodová Vareková R, Koca J, Otyepka M. MOLEonline: a web-based tool for analyzing channels, tunnels and pores (2018 update). Nucleic Acids Res. 2018 Jul 2;46(W1):W368-W373).

Supplementary Table S3. TIM activity determination from *E. coli* BL21-CodonPlus-RIL cells with WT and N16D HsTIM or without gene insert.

***E. coli* BL21-CodonPlus-RIL**	**Condition**	**Enzyme Activity (%)**	**Enzyme activity (µmol/min mg)**
WT	Control*	100	286 ± 6
	+ Omeprazole	93 ± 5	267.5 ± 19.5
N16D	Control*	100	19.2 ± 0.6
	+ Omeprazole	51 ± 4	9.8± 0.5
**	Control*	100	11.7± 1.5
	+ Omeprazole	87 ± 2	10.1± 1.6

* cells without drug. ** cells without WT or N16D gene insert. Values correspond to the endogenous bacterial TIM activity.

Supplementary Table S4. Characteristics of the thiol-reactive compounds

Name	Molecular Formula	Molecular Mass (Daltons)	Chemical Structure
methyl-methanethiosulfonate<br>**MMTS**	$C_2H_6O_2S$	126.198	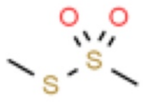
sodium 2-[(methylsulfonyl)sulfanyl] ethanesulfonate<br>**MTSES**	$C_3H_7NaO_5S_3$	242.269	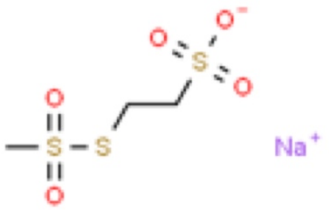
5,5′-dithiobis-(2-nitrobenzoic acid)<br>**DTNB**	$C_{14}H_8N_2O_8S_2$	396.352	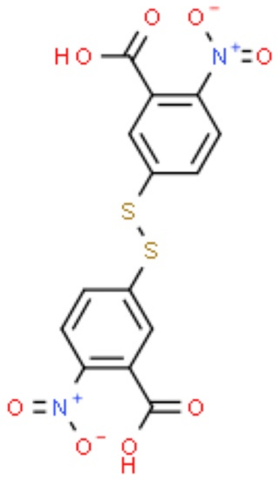
(5-methoxy-2-[[(4-methoxy-3,5-dimethyl-2-pyridinyl) methyl] sulfinyl]-1H-benzimidazole)<br>**Omeprazole**	$C_{17}H_{19}N_3O_3S$	345.416	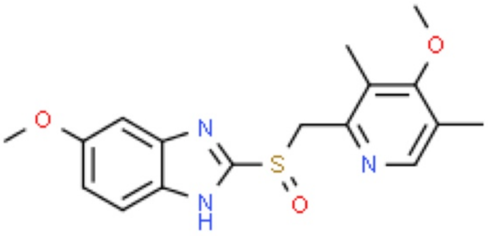

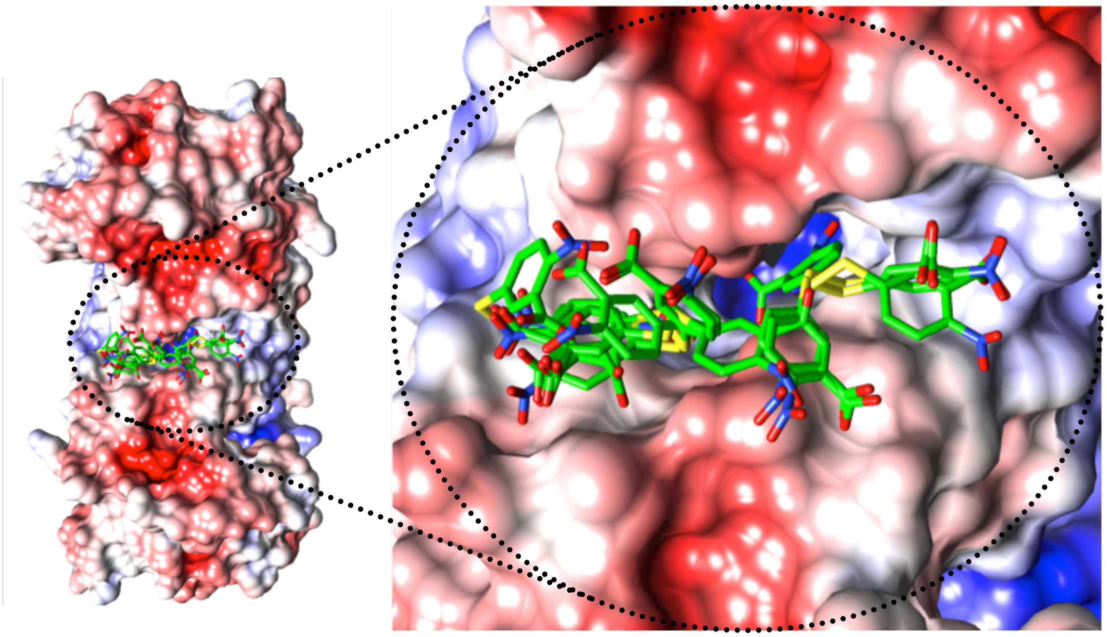

Supplementary Figure S1. Docking of DTNB and electrostatic potential surface of HsTIM WT structure. The figures are an ensemble of docking and the electrostatic potential surface results. As seen in WT, the different conformers of DTNB were incorporated superficially in the interface of HsTIM structure. Color codes represent electrostatic potential surface energy values of -5.0 (■) and +5.0 (■). Figures were modeled with the molecular graphics UCSF Chimera package (Pettersen, E.F., Goddard, T.D., Huang, C.C., Couch, G.S., Greenblatt, D.M., Meng, E.C., and Ferrin, T.E. "UCSF Chimera - A Visualization System for Exploratory Research and Analysis." *J. Comput. Chem.* **25**(13):1605-1612 (2004).

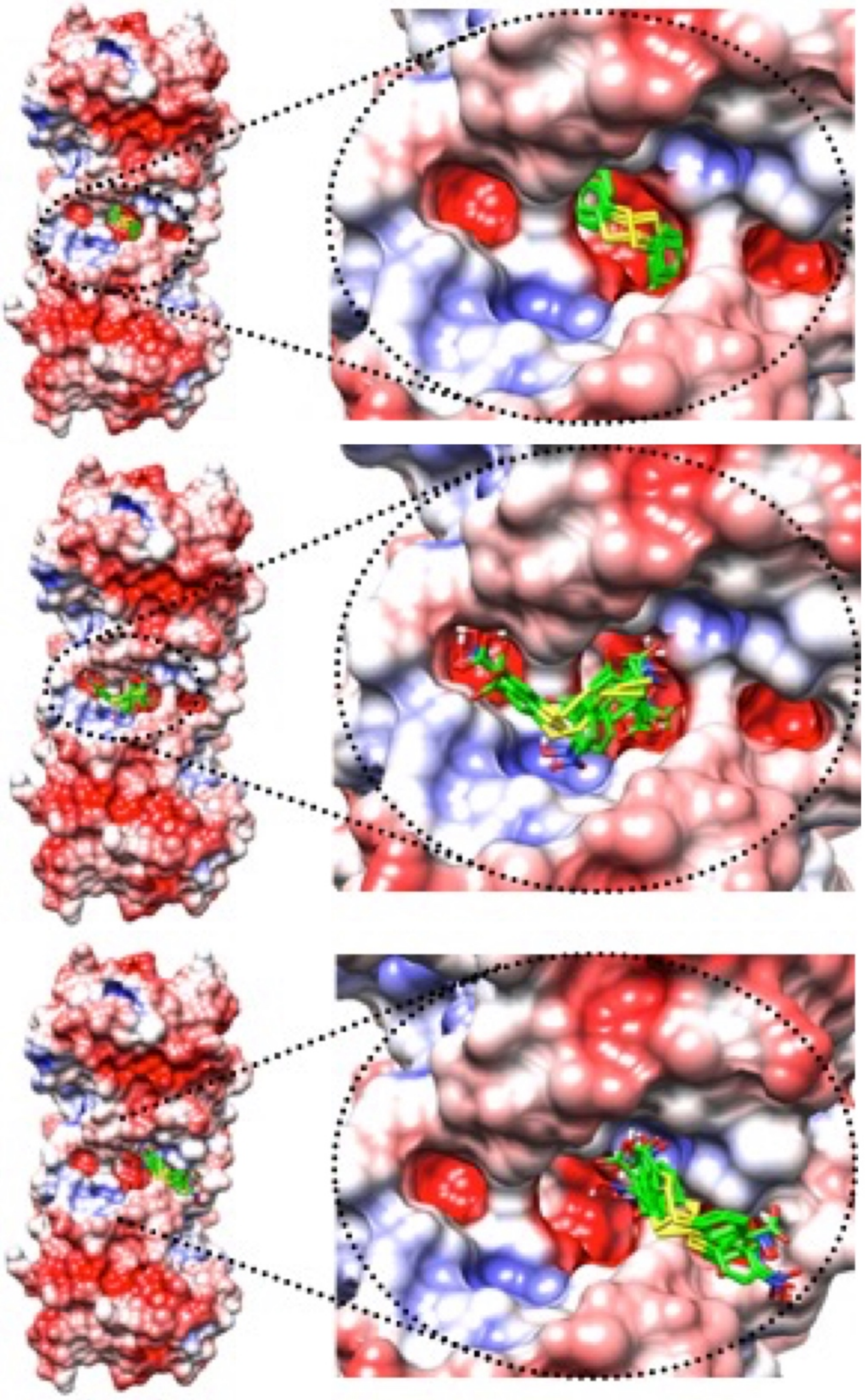

Supplementary Figure S2. Docking of DTNB and electrostatic potential surface of HsTIM N16D structures. The figures are an ensemble of docking and the electrostatic potential surface results. As seen in N16D, major number of conformers of DTNB were docked in the same region (unlike the WT, where the DTNB conformers were docked superficially). Color codes represent electrostatic potential surface energy values of -5.0 (■) and +5.0 (■). Figures were modeled with the molecular graphics UCSF Chimera package (Pettersen, E.F., Goddard, T.D., Huang, C.C., Couch, G.S., Greenblatt, D.M., Meng, E.C., and Ferrin, T.E. "UCSF Chimera - A Visualization System for Exploratory Research and Analysis." *J. Comput. Chem.* **25**(13):1605-1612 (2004).

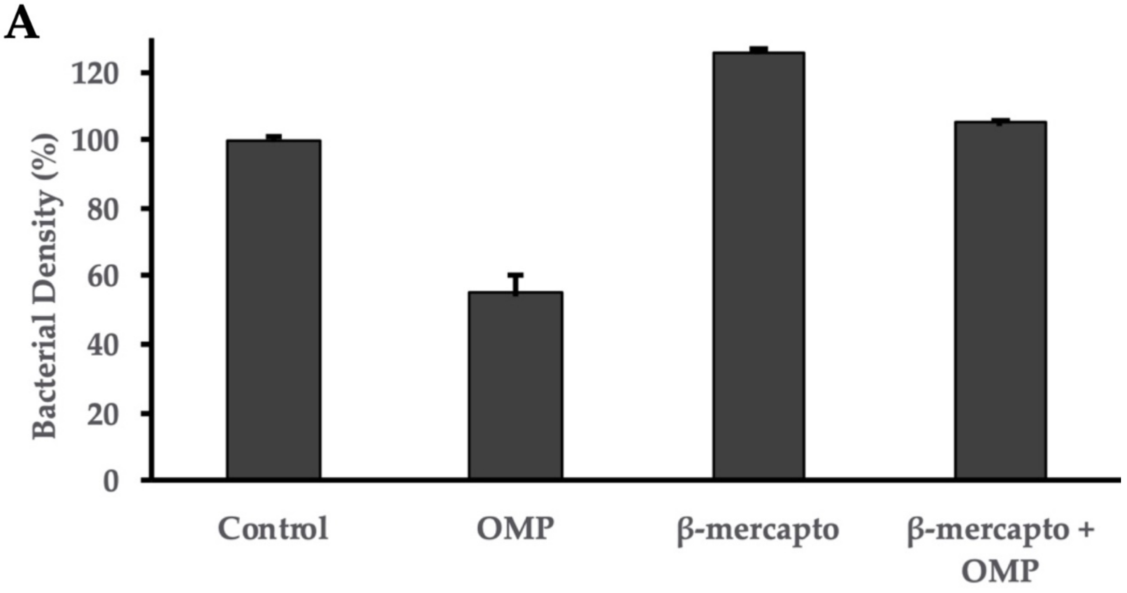

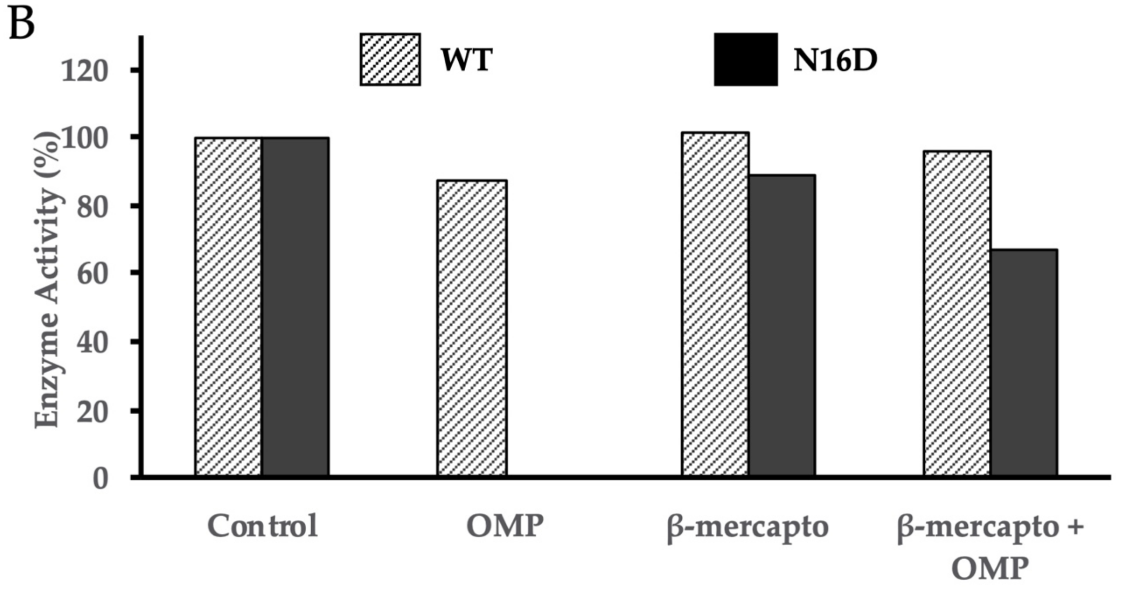

Supplementary Figure S3. Scavenger effect of β-mercaptoethanol (β-mercapto) on omeprazole (OMP) sulfenamide. A) shows the bactericidal activity of OMP on *E. coli* Δ*tim* cells complemented with N16D and the reduction of such activity by using β-mercaptoethanol (β-mercapto + OMP). B) shows the effect of OMP on the enzyme activity of recombinant WT and N16D HsTIM. While WT HsTIM is almost unaffected neither by OMP nor by β-mercaptoethanol, N16D HsTIM activity is unaffected by β-mercaptoethanol (β-mercapto) but completely depleted by OMP; nevertheless, when β-mercaptoethanol is added (β-mercapto + OMP) enzyme activity is recovered. Recombinant WT HsTIM control showed enzyme activity of 4684 μmol/min/mg, whereas N16D HsTIM control showed 640 μmol/min/mg.

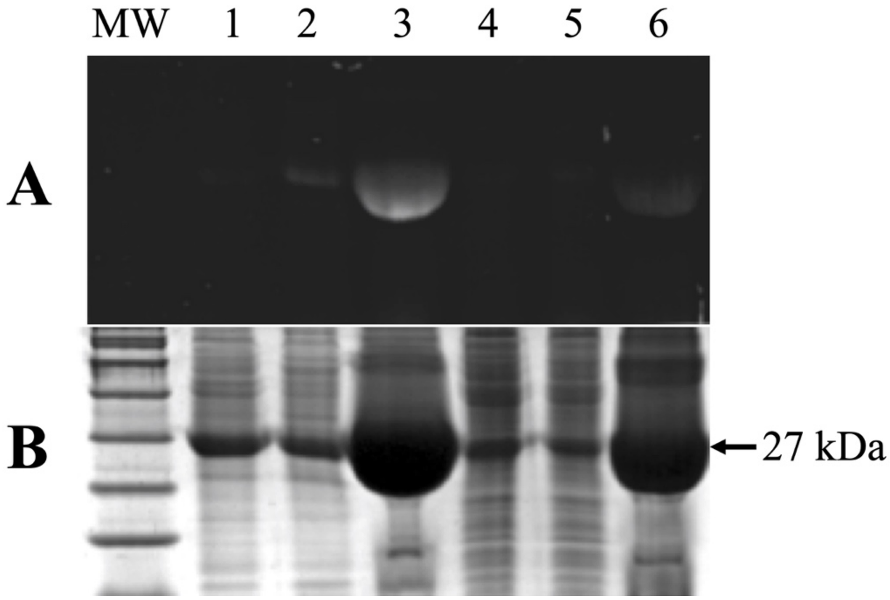

Supplementary Figure 4. Fluorescence of HsTIM-omeprazole adducts. A) shows the SDS-PAGE previously to be staining and photographed in a UV transilluminator. B) shows the same gel stained with Coomassie brilliant blue. MW: Molecular weight marker (BioRad broad range). Total proteins extract (30 µg) from *E. coli* BL21-CodonPlus (DE3)-RIL overexpressing N16D or WT HsTIM previously incubated in absence (lanes 1and 4) or presence (lanes 2 and 5) of 0.75 mM omeprazole, respectively. Lanes 3 and 6 show partially purified N16D and WT HsTIM (50 µg, each) coming from the cells previously incubated with 0.75 mM omeprazole. Arrow shows the molecular mass of HsTIM monomer.
